# Knowledge, attitudes, and practices toward over-the-counter antipyretics among fever patients: a cross-sectional study in the context of a policy change KAP of OTC antipyretics

**DOI:** 10.3389/fpubh.2023.1267171

**Published:** 2023-11-10

**Authors:** Yan Zhang, Shuchang Liang, Tao Zhu

**Affiliations:** ^1^Department of Infectious Diseases, People’s Hospital of Dongxihu District, Wuhan, Hubei, China; ^2^Department of Pediatrics, Yichang Traditional Chinese Medicine Hospital, Yichang, Hubei, China

**Keywords:** over-the-counter, antipyretics, fever patients, knowledge, attitudes, practices, cross-sectional study

## Abstract

**Background:**

On January 8, 2023, a change in the control policy for COVID-19 was implemented in China, whereby patient self-management of fever typically entails the utilization of over-the-counter fever-reducing medications.

**Objective:**

This study aimed to investigate the knowledge, attitudes, and practices (KAP) toward over-the-counter (OTC) antipyretics among fever patients.

**Methods:**

This cross-sectional study was conducted between October 2022 and February 2023 at author’s hospital in Wuhan, China, among fever patients on OTC antipyretics, using a self-administered questionnaire.

**Results:**

A total of 481 valid questionnaires were collected, with the age of 36.05 ± 12.10 years, including 240 (49.90%) males, and 209 (43.45%) collected before policy change. The knowledge, attitudes, precautions for medication administration and decision-making practices scores were 6.86 ± 3.30 (possible range: 0–12), 16.67 ± 2.46 (possible range: 7–35), 29.98 ± 5.41 (possible range: 7–35) and 27.87 ± 1.28 (possible range: 8–40), respectively. The multivariable logistic regression analysis showed that knowledge (OR = 0.83, 95%CI: 0.81–0.92, *p* < 0.001) was independently associated with positive attitude. Knowledge (OR = 1.41, 95%CI: 1.28–1.56, *p* < 0.001), attitude (OR = 0.87, 95%CI: 0.79–0.95, *p* = 0.004), suburban (OR = 0.45, 95%CI: 0.23–0.88, *p* = 0.019) were independently associated with proactive precautions for medication administration practices. Knowledge (OR = 1.14, 95%CI: 1.07–1.22, *p* < 0.001), attitude (OR = 0.90, 95%CI: 0.82–0.98, *p* = 0.018), responding after policy change, 2023 (OR = 1.70, 95%CI: 1.10–2.63, *p* = 0.016) were independently associated with proactive decision making practices.

**Conclusion:**

Fever patients had moderate knowledge, negative attitude, proactive precautions for medication administration practices, moderate decision-making practices. After the policy change, there was a significant increase in knowledge regarding medication administration precautions and decision-making.

## Introduction

Antipyretics are commonly used to relieve fever, a common symptom of many illnesses (1). Over-the-counter (OTC) antipyretics are non-prescription medications, readily available to individuals suffering from fever, thus ensuring convenient accessibility. Antipyretics are available in various forms, including tablets, capsules, liquids, and suppositories, and contain active ingredients such as acetaminophen (paracetamol), ibuprofen, and aspirin (2). However, the indiscriminate use of OTC antipyretics can have adverse effects and mask underlying symptoms, leading to delayed diagnosis and treatment (3–5). Therefore, it is important for patients to understand the appropriate use and potential risks of OTC antipyretics.

Knowledge, attitudes, and practices (KAP) studies serve as essential tools for understanding how populations acquire and process information, and how this information shapes their behavior. These studies offer valuable insights into public health awareness, policy implementation, and the effectiveness of health promotion campaigns (6, 7). Numerous KAP studies have uncovered a significant lack of knowledge and poor practices about antipyretics usage or fever management among health professionals, parents, and caregivers worldwide (8–12). According to a recent survey, antipyretic analgesics rank at the top of OTC drugs that have been purchased and utilized. Among respondents, 58.57% have purchased and used antipyretics (13). However, there is a lack of KAP assessments focused on Chinese fever patients.

The COVID-19 pandemic has contributed to a growing trend of self-medication and increased OTC use of antipyretics, which may lead to incorrect dosing and potential drug interactions (14, 15). Therefore, it is crucial to understand the KAP of fever patients toward OTC antipyretics to ensure the safe and effective use of these medications. In light of the reclassification and subsequent removal of COVID-19 from quarantine management in China on January 8th, 2023, this policy change may potentially impact individuals’ attitudes and behaviors toward medication use (16, 17). Specifically, if people believe that COVID-19 is no longer a serious infectious disease, they could be more likely to purchase and use OTC antipyretics to relieve symptoms. However, this increase in demand could lead to improper usage of OTC antipyretics, such as exceeding recommended dosages or mixing different drugs, thereby increasing the risk of adverse effects (18). Therefore, analyzing KAP for OTC antipyretics before and after this policy change could be an interesting area of study.

This study aimed to examine the KAP of fever patients in China regarding OTC antipyretics in light of the policy change.

## Methods

### Study design and participants

This cross-sectional study was conducted between October 2022 and February 2023 at author’s hospital in Wuhan, China, and included fever patients. The inclusion criteria were: (1) exhibiting a febrile condition characterized by a body temperature of 37.5 degrees Celsius; (2) autonomous understanding and expression ability; and (3) willingness to participate. Exclusion criteria were: (1) age below 18 or above 70 years. The study was approved by the Medical Ethics Committee of the People’s Hospital of Dongxihu District, Wuhan, Hubei, and all participants provided informed consents before completing the questionnaire.

We employed a convenience sampling method to select participants. We determined the required sample size using the following formula: $n={\left(\frac{Z_{1-\alpha /2}}{\delta }\right)}^{2}\times p\times \left(1-p\right)$, as previously described (19). In this formula, “*n*” represents the required sample size, “*α*” represents the type I error, which is typically set at 0.05, $Z_{1-\alpha /2}$ = 1.96, “*δ*” represents the allowable error, usually set at 0.05, and “*p*” is set at 0.5. Setting “*p*” at 0.5 maximizes the required sample size, ensuring adequate statistical power. The required sample size “*n*” was determined to be 384. Considering the estimated questionnaire response rate of 80%, our final plan was to collect 480 valid questionnaires.

### Procedures

According to the Guidelines for Evidence-Based Diagnosis and Management of Acute Fever Without a Source in Children Aged 0–5 Years (20), the Expert Consensus on Diagnosis and Treatment of Fever of Unknown Origin (21), the Expert Consensus on Rational Use of Antipyretics and Analgesics in Treating Fever in Children (22), the Guidelines for Symptomatic Management of Fever in Children, and a systematic literature review (23), a four-dimensional questionnaire was developed and reviewed by five experts (three in infectious diseases and two in pediatrics). The initial version of the questionnaire exhibited inaccuracies, primarily within the practice dimension. It placed greater emphasis on the accessibility of medicines, rather than capturing medication-related decisions made by patients. Following expert feedback, the research team incorporated several modifications to the questionnaire. Notably, the practice dimension was divided into two distinct sections to enhance the evaluation of patient medication decisions. The pre-test involved administering 31 copies of the questionnaire, and the results showed a high level of internal consistency, with a Cronbach’s α coefficient of 0.896, indicating good internal consistency.

The final version of the questionnaire included four dimensions: (1) Demographic characteristics included gender, age, type of residence, education, occupation, marital status, presence of children, medical insurance, and included an item to record the date of response; (2) Knowledge dimension included 12 questions, and scored as 1 for correct answer and 0 for incorrect or unclear answer; (3) Attitude dimension, which comprised 7 questions measured on a 5-point Likert scale ranging from very positive (5 points) to very negative (1 point); and (4) Practice dimension, which was split into two sub-sections: precautions for medication administration (7 questions) measured on a 5-point Likert scale ranging from always (5 points) to never (1 point), and decision-making (8 questions) scored as 5 for correct choices and 0 for incorrect choices. A score above 75% of the total score of each dimention is considered good, while a score between 50 and 75% is considered moderate, and a score below 50% is considered poor, as previously described (24).

Five trained research assistants were responsible for guiding the participants through the questionnaire completion process. They provided verbal instructions and clarified any questions that participants had, but they refrained from influencing the participants’ choices. The assistants had received training on how to interact with patients and had practiced using the questionnaire in a pilot study involving 31 patients. They also explained the study’s purpose to the participants and emphasized the importance of their participation. To minimize the burden on the participants, the questionnaire was designed to be completed in approximately 20 min. Health promotion activities, such as educational materials and demonstrations on the OTC antipyretics, were conducted after the responses were collected to show appreciation for the participants’ time and effort. An online questionnaire was created using the WeChat-based Wen Juan Xing (WJX) platform^1^ in China, and a quick response (QR) code was generated for data collection via WeChat. Patients could easily scan the QR code to access and complete the questionnaire using their smartphones. To ensure the quality and completeness of the responses, each IP address was limited to a single submission, and all questions were made mandatory. For patients who were unable to complete the questionnaire via mobile phone, a paper version was provided for easier completion. After the data collection, the research team members checked all submitted questionnaires for completeness, internal consistency, and reasonableness. Any missing or inconsistent responses were reviewed with the participants to clarify their answers.

### Statistical analysis

Statistical analysis was performed using Stata 17.0 software (Stata Corporation, College Station, TX, USA). Continuous data were expressed as mean ± standard deviation (SD) and compared by *t*-test or one-way analysis of variance (ANOVA). The categorical data were presented as *n* (%) and compared by the chi-square test. Multivariable logistic regression analyses were employed. A 70% distribution of scores for KAP was used as the cut-off values. The distribution of the data was used to select the cut-off values because the data distribution was too skewed. Covariance tests for multivariate regression variable inclusion were conducted using variance inflation factors (VIF) for demographic profile variables. All variables in the final model had a VIF < 3 and a KAP score. A two-sided *p* < 0.05 was considered statistically significant.

## Results

### Demographic characteristics

A total of 496 questionnaires were collected. After a thorough review of the responses, 3 participants who did not provide informed consent and 12 participants who provided incorrect age information were excluded, resulting in 481 valid questionnaires (96.98%). The patients had the age of 36.05 ± 12.10 years, with 240 (49.90%) being male, and 209 (43.45%) collected before policy change (Table 1).

**Table 1:** Baseline characteristics and KAP scores.

Variables	*N* (%)	Knowledge score		Attitude score		Practice score (precautions for medication administration)		Practice score (decision making)	
		Mean ± SD	*p*	Mean ± SD	*p*	Mean ± SD	*p*	Mean ± SD	*p*
*Total*	481	6.86 ± 3.30		16.67 ± 2.46		29.98 ± 5.41		27.87 ± 1.28	
*Point-in-time*			0.002		0.059		0.038		<0.001
Before policy change	209 (43.45)	6.33 ± 3.46		16.91 ± 2.51		29.39 ± 5.85		27.61 ± 1.34	
After policy change	272 (56.55)	7.27 ± 3.12		16.48 ± 2.41		30.42 ± 5.02		28.06 ± 1.19	
*Gender*			0.019		0.333		0.167		0.834
Male	240 (49.90)	6.50 ± 3.69		16.56 ± 2.49		29.63 ± 5.75		27.88 ± 1.30	
Female	241 (50.10)	7.21 ± 2.83		16.78 ± 2.43		30.32 ± 5.05		27.85 ± 1.25	
*Age, year*	36.05 ± 12.10								
*Type of residence*			0.008		0.660		< 0.001		0.183
Rural	110 (22.87)	6.25 ± 3.57		16.61 ± 2.53		29.14 ± 5.79		27.67 ± 1.27	
Urban	284 (59.04)	7.25 ± 3.10		16.62 ± 2.47		30.73 ± 4.86		27.94 ± 1.30	
Suburban	87 (18.09)	6.36 ± 3.45		16.89 ± 2.36		28.56 ± 6.20		27.89 ± 1.21	
*Education*			0.039		0.516		0.046		0.013
Primary School and below	20 (4.16)	5.10 ± 4.36		15.95 ± 1.73		27.75 ± 6.52		27.20 ± 1.24	
Middle School	68 (14.14)	6.32 ± 3.68		16.60 ± 2.47		28.74 ± 6.41		27.62 ± 1.30	
High School/Technical Secondary School	110 (22.87)	7.02 ± 3.32		16.48 ± 2.44		30.22 ± 5.07		27.79 ± 1.15	
Junior College / Undergraduate	260 (54.05)	6.98 ± 3.08		16.80 ± 2.49		30.23 ± 5.22		27.98 ± 1.33	
Postgraduate and above	23 (4.78)	7.83 ± 3.01		16.83 ± 2.76		31.52 ± 3.96		28.26 ± 0.92	
*Occupation*			< 0.001		0.039		0.059		0.027
Management personnel/Professional technicians	104 (21.62)	7.92 ± 2.83		16.13 ± 2.43		31.15 ± 4.89		28.08 ± 1.21	
Employees	117 (24.32)	6.92 ± 3.12		16.67 ± 2.47		29.85 ± 4.68		27.84 ± 1.46	
Service-related personnel/Production and transport personnel	97 (20.17)	6.14 ± 3.55		16.66 ± 2.46		29.14 ± 6.35		27.56 ± 1.30	
Other	163 (33.89)	6.56 ± 3.42		17.02 ± 2.43		29.80 ± 5.54		27.94 ± 1.14	
*Marital status*			0.009		0.021		0.766		0.843
Unmarried	137 (28.48)	6.28 ± 3.43		17.14 ± 2.54		29.72 ± 5.24		27.89 ± 1.29	
Married	323 (67.15)	7.17 ± 3.18		16.51 ± 2.41		30.06 ± 5.52		27.85 ± 1.27	
Other	21 (4.37)	5.76 ± 3.71		16.05 ± 2.33		30.43 ± 5.09		28.00 ± 1.41	
*Children brought up*			0.005		< 0.001		0.495		0.543
Yes	323 (67.15)	7.15 ± 3.17		16.41 ± 2.40		30.09 ± 5.54		27.84 ± 1.26	
No	158 (32.85)	6.26 ± 3.49		17.20 ± 2.51		29.73 ± 5.15		27.92 ± 1.32	
*Medical insurance*			0.143		0.868		0.244		0.529
Yes	464 (96.47)	6.90 ± 3.28		16.66 ± 2.46		30.03 ± 5.34		27.86 ± 1.28	
No	17 (3.53)	5.71 ± 3.75		16.76 ± 2.66		28.47 ± 7.05		28.06 ± 1.25	

### Knowledge, attitudes, and practices

**Figure 1:**
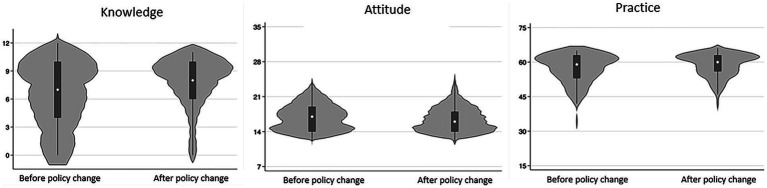
Comparison of KAP scores among patients participating before and after policy change.

The knowledge, attitudes, precautions for medication administration and decision-making practices scores were 6.86 ± 3.30 (possible range: 0–12), 16.67 ± 2.46 (possible range: 7–35), 29.98 ± 5.41 (possible range: 7–35) and 27.87 ± 1.28 (possible range: 8–40), respectively, indicating moderate knowledge, negative attitude, proactive precautions for medication administration practices, moderate decision-making practices (Table 1). Higher scores observed among participants who responded after policy change (*p* = 0.002 for knowledge, *p* = 0.038 for precautions for medication administration, *p* < 0.001 for decision making), females (*p* = 0.019 for knowledge), urban residents (*p* = 0.008 for knowledge, *p* < 0.001 for precautions for medication administration), those with higher education (*p* = 0.039 for knowledge, *p* = 0.046 for precautions for medication administration, *p* = 0.013 for decision making), management personnel/professional technicians (*p* < 0.001 for knowledge, *p* = 0.027 for decision making), married individuals (*p* = 0.009 for knowledge, *p* = 0.021 for attitudes), and those with children (*p* = 0.005 for knowledge, *p* < 0.001 for attitudes; Table 1; Figure 1).

Patients demonstrated varying levels of accurate knowledge about antipyretics. The highest correct score was for the understanding that antipyretics are recommended for fevers above 38.5°C, with 369 participants (76.72%) answering correctly. On the other hand, the lowest correct score was for the guideline that antipyretics should be taken every 6–8 h, at least 6 h apart, and not exceeding 3 times daily, with only 23 patients (4.78%) providing the correct response. The most positive attitude was toward the desire for education from physicians about antipyretics, with 61.33% of respondents indicating a very positive attitude and 31.19% indicating a positive attitude. The most negative attitude was toward physical cooling being preferred over antipyretics, with 7.28% of respondents indicating a negative attitude and 0.83% indicating a very negative attitude. The participants generally practice well in handling and storing medications, such as reading instructions carefully and retaining outer packaging (58.63% for always and 26.61% for often). However, they do not perform as well when it comes to making correct decisions about medication usage in specific situations, such as avoiding combining acetaminophen with ibuprofen for fever reduction in children (45.32% participants choose the wrong answer) or using ibuprofen to treat fever in children diagnosed with chickenpox (50.73% participants choose the wrong answer; Table 2).

**Table 2:** Questionnaires.

Knowledge	Correct rate *n* (%)
1. Antipyretics are recommended for fevers above 38.5°C.	369 (76.72)
2. For fevers below 38.5°C, maintaining water and electrolyte balance is sufficient, as antipyretic treatment may affect diagnosis and prognosis.	361 (75.05)
3. Paracetamol, Panadol, and Tylenol all contain the single effective ingredient “acetaminophen.”	266 (55.30)
4. Common antipyretics include ibuprofen, acetaminophen, diclofenac, aspirin, indomethacin, Metamizole Sodium Tablets, and aminopyrine.	344 (71.52)
5. Most antipyretics begin working within 2 h, with an average onset of 1 h after taking the medication.	349 (72.56)
6. Antipyretics should generally be taken every 6–8 h, at a minimum interval of 6 h, and no more than 3 times a day. (Incorrect)	23 (4.78)
7. Ibuprofen is commonly used for children’s fever but is contraindicated in pregnant women.	332 (69.02)
8. Alternating acetaminophen and ibuprofen is more effective for relieving fever symptoms. (Incorrect)	145 (30.15)
9. Aspirin and acetaminophen relieve fever, headache, myalgia, and arthralgia, but may cause platelet and gastrointestinal side effects.	308 (64.03)
10. Acetaminophen and ibuprofen are the only recommended antipyretics for children.	240 (49.90)
11. In China, metamizole sodium tablets are not the first choice for antipyretics.	316 (65.70)
12. Honey can reduce acetaminophen’s effectiveness, increasing adverse reaction risks; they should not be taken together.	246 (51.14)

Attitude	Very positive	Positive	Neutral	Negative	Very negative
1. The variety and dosage forms of antipyretics in clinics are confusing. (N)	198 (41.16)	124 (25.78)	135 (28.07)	16 (3.33)	8 (1.66)
2. Distinguishing between types and names of antipyretics is challenging. (N)	195 (40.54)	164 (34.1)	102 (21.21)	12 (2.49)	8 (1.66)
3. Education from physicians about antipyretics is desired. (P)	295 (61.33)	150 (31.19)	32 (6.65)	0 (0)	4 (0.83)
4. Physical cooling is preferred over antipyretics.	197 (40.96)	136 (28.27)	109 (22.66)	35 (7.28)	4 (0.83)
5. Concerns exist about antipyretics causing liver and kidney damage. (N)	208 (43.24)	170 (35.34)	87 (18.09)	13 (2.7)	3 (0.62)
6. Worries about adverse effects, such as gastrointestinal reactions, from antipyretics are present. (N)	206 (42.83)	166 (34.51)	95 (19.75)	11 (2.29)	3 (0.62)
7. Vulnerable populations, including the older adult, pregnant women, children, and those with drug allergies, should be more cautious when choosing antipyretics. (P)	331 (68.81)	111 (23.08)	37 (7.69)	0 (0)	2 (0.42)

Practice(precautions for medication administration)	Always	Often	Sometimes	Rarely	Never
1. Carefully read the instructions before using the medicine, and pay close attention to the drug specifications. (P)	282 (58.63)	128 (26.61)	56 (11.64)	12 (2.49)	3 (0.62)
2. Be cautious with cold and flu medications containing antipyretic ingredients; avoid taking multiple doses. (P)	252 (52.39)	111 (23.08)	78 (16.22)	32 (6.65)	8 (1.66)
3. Store the medicine according to the conditions specified in the instructions (e.g., in a shaded, sealed, and cool location). (P)	272 (56.55)	119 (24.74)	60 (12.47)	23 (4.78)	7 (1.46)
4. Retain the outer packaging while storing the medicine. (P)	288 (59.88)	91 (18.92)	68 (14.14)	25 (5.2)	9 (1.87)
5. Minimize contamination during use (e.g., use non-disposable, specialized measuring utensils for fluids; disinfect and dry utensils before they come into contact with medications). (P)	247 (51.35)	111 (23.08)	80 (16.63)	30 (6.24)	13 (2.7)
6. Increase fluid intake during the fever relief process to prevent collapse and shock. (P)	277 (57.59)	125 (25.99)	61 (12.68)	10 (2.08)	8 (1.66)
7. Seek medical attention promptly if symptoms do not significantly improve or disappear after 3 days of symptomatic treatment. (P)	278 (57.8)	111 (23.08)	61 (12.68)	19 (3.95)	12 (2.49)

Practice(decision making)	Correct	Wrong
8. Avoid combining acetaminophen with ibuprofen for fever reduction in children. (N)	218 (45.32)	263 (54.68)
9. Routinely administer acetaminophen prophylactically before and after vaccinating children. (N)	274 (56.96)	207 (43.04)
10. Refrain from using aspirin, diclofenac, and ibuprofen during pregnancy or remind pregnant women in the family. (P)	341 (70.89)	140 (29.11)
11. Choose acetaminophen to alleviate fever in individuals with a history of gastric or duodenal ulcers or gastrointestinal bleeding. (P)	237 (49.27)	244 (50.73)
12. Use acetaminophen for fever relief and analgesia in cases of cardiovascular disease. (P)	233 (48.44)	248 (51.56)
13. Use ibuprofen to treat fever in children diagnosed with chickenpox. (N)	244 (50.73)	237 (49.27)
14. Decrease the dosage of antipyretics appropriately when administering them to older adult individuals. (P)	373 (77.55)	108 (22.45)
15. Use antipyretics cautiously in patients with liver or kidney dysfunction. (P)	421 (87.53)	60 (12.47)

P represents “positive,” indicating responses ranging from “strongly agree” to “strongly disagree” with a score of 5–1 points. N represents “negative,” indicating responses ranging from “strongly agree” to “strongly disagree” with a score of 1–5 points.

### Multivariable logistic regression analysis

The multivariable logistic regression analysis showed that knowledge (OR = 0.83, 95%CI: 0.81–0.92, *p* < 0.001) was independently associated with positive attitude. Knowledge (OR = 1.41, 95%CI: 1.28–1.56, *p* < 0.001), attitude (OR = 0.87, 95%CI: 0.79–0.95, *p* = 0.004), suburban (OR = 0.45, 95%CI: 0.23–0.88, *p* = 0.019) were independently associated with precautions for medication administration. Knowledge (OR = 1.14, 95%CI: 1.07–1.22, *p* < 0.001), attitude (OR = 0.90, 95%CI: 0.82–0.98, *p* = 0.018), responding after policy change (OR = 1.70, 95%CI: 1.10–2.63, *p* = 0.016) were independently associated with proactive practice decision making (Table 3).

**Table 3:** Multivariate logistic regression analysis.

		OR (95% CI)	*p*
Knowledge	*Point-in-time*		
	Before policy change	Ref.	
	After policy change	1.16 (0.78, 1.73)	0.468
	*Gender*		
	Male	Ref.	
	Female	0.92 (0.62, 1.37)	0.685
	*Type of residence*		
	Urban	Ref.	
	Rural	0.79 (0.45, 1.38)	0.401
	Suburban	0.83 (0.48, 1.44)	0.507
	*Education*		
	Junior College/Undergraduate	Ref.	
	Primary School and below	0.98 (0.31, 3.08)	0.969
	Middle School	1.16 (0.62, 2.19)	0.641
	High School/Technical Secondary School	1.41 (0.86, 2.31)	0.177
	Postgraduate and above	1.06 (0.42, 2.69)	0.907
	*Occupation*		
	Employees	Ref.	
	Management personnel/Professional technicians	1.46 (0.81, 2.64)	0.211
	Service-related personnel/Production and transport personnel	0.73 (0.40, 1.36)	0.323
	Other	0.88 (0.52, 1.48)	0.620
Attitude	*K*	0.86 (0.81, 0.92)	< 0.001
	*Point-in-time*		
	Before policy change	Ref.	
	After policy change	0.83 (0.56, 1.23)	0.353
	*Gender*		
	Male	Ref.	
	Female	1.31 (0.88, 1.95)	0.190
	*Type of residence*		
	Urban	Ref.	
	Rural	0.98 (0.57, 1.67)	0.939
	Suburban	0.68 (0.40, 1.18)	0.175
	*Education*		
	Junior College/Undergraduate	Ref.	
	Primary School and below	0.49 (0.16, 1.51)	0.213
	Middle School	0.74 (0.40, 1.36)	0.335
	High School/Technical Secondary School	0.75 (0.46, 1.24)	0.265
	Postgraduate and above	2.09 (0.79, 5.48)	0.135
	*Occupation*		
	Employees	Ref.	
	Management personnel/Professional technicians	0.60 (0.32, 1.15)	0.125
	Service-related personnel/Production and transport personnel	1.11 (0.61, 2.01)	0.726
	Other	1.44 (0.87, 2.40)	0.160
Practice frequency	*K*	1.41 (1.28, 1.56)	< 0.001
	*A*	0.87 (0.79, 0.95)	0.004
	*Point-in-time*		
	Before policy change	Ref.	
	After policy change	0.80 (0.51, 1.25)	0.321
	*Gender*		
	Male	Ref.	
	Female	0.87 (0.56, 1.36)	0.542
	*Type of residence*		
	Urban	Ref.	
	Rural	0.81 (0.44, 1.50)	0.497
	Suburban	0.45 (0.23, 0.88)	0.019
	*Education*		
	Junior College/Undergraduate	Ref.	
	Primary School and below	0.95 (0.25, 3.58)	0.945
	Middle School	1.14 (0.54, 2.37)	0.735
	High School/Technical Secondary School	1.21 (0.69, 2.12)	0.504
	Postgraduate and above	0.79 (0.27, 2.30)	0.666
	*Occupation*		
	Employees	Ref.	
	Management personnel/Professional technicians	1.30 (0.66, 2.56)	0.444
	Service-related personnel/Production and transport personnel	1.55 (0.77, 3.11)	0.216
	Other	1.68 (0.92, 3.08)	0.094
Practice decision making	*K*	1.14 (1.07, 1.22)	< 0.001
	*A*	0.90 (0.82, 0.98)	0.018
	*Point-in-time*		
	Before policy change	Ref.	
	After policy change	1.70 (1.10, 2.63)	0.016
	*Gender*		
	Male	Ref.	
	Female	0.86 (0.55, 1.33)	0.497
	*Type of residence*		
	Urban	Ref.	
	Rural	0.94 (0.53, 1.68)	0.842
	Suburban	1.25 (0.68, 2.27)	0.472
	*Education*		
	Junior College/Undergraduate	Ref.	
	Primary School and below	0.67 (0.22, 2.04)	0.479
	Middle School	0.84 (0.44, 1.62)	0.611
	High School / Technical Secondary School	0.93 (0.54, 1.59)	0.787
	Postgraduate and above	1.41 (0.42, 4.71)	0.581
	*Occupation*		
	Employees	Ref.	
	Management personnel/Professional technicians	1.46 (0.74, 2.88)	0.281
	Service-related personnel/Production and transport personnel	0.78 (0.43, 1.44)	0.433
	Other	1.81 (1.03, 3.18)	0.039

### Pearson correlation analysis

The results of Pearson correlation analysis indicated that knowledge exhibited a negative correlation with attitudes (*r* = −0.231, *p* < 0.001). Conversely, knowledge showed positive correlations with total practices (*r* = 0.535, *p* < 0.001), precautions for medication administration (*r* = 0.523, *p* < 0.001), and decision making (*r* = 0.234, *p* < 0.001). Furthermore, attitude displayed negative correlations with both total practices (*r* = −0.183, *p* < 0.001) and precautions for medication administration (*r* = −0.183, *p* < 0.001; Table 4).

**Table 4:** Pearson correlation analysis.

	Knowledge	Attitude	Practice
Total			
Knowledge	1		
Attitude	−0.231 (*p* < 0.001)	1	
Practice	0.535 (*p* < 0.001)	−0.183 (*p* < 0.001)	1
Practice – precautions for medication administration			
Knowledge	1		
Attitude	−0.231 (*p* < 0.001)	1	
Practice	0.523 (*p* < 0.001)	−0.183 (*p* < 0.001)	1
Practice – decision making			
Knowledge	1		
Attitude	−0.231 (*p* < 0.001)	1	
Practice	0.234 (*p* < 0.001)	−0.061 (*p* < 0.186)	1

## Discussion

Overall, fever patients displayed moderate levels of knowledge, negative attitudes, proactive precautions for medication administration practices, and moderate decision-making practices. However, subsequent to the policy change, there was a noteworthy enhancement in knowledge regarding medication administration precautions and decision-making among fever patients.

This study found varying knowledge and attitudes among Chinese fever patients on OTC antipyretics usage, with practice frequency being relatively high, and highlighted the need for improved education and guidance to promote better decision-making. Participants in this study scored an average of 57.17% in knowledge, 47.63% in attitudes, and 85.66% in practice frequency, while their decision-making practice scored 69.68%. This suggests that while practices (decision making) was relatively high, there were gaps in knowledge and attitudes. Similarly, other studies revealed that parents and pediatricians held some misconceptions and incorrect practices in fever management, emphasizing the need for targeted educational interventions (25–29).

In this study, fever patients demonstrated a good understanding of the recommended usage of antipyretics for fevers above 38.5°C, common antipyretic medications, and the average onset time for these medications to start working, with correctness rates exceeding 70%. However, they showed limited knowledge about dosing intervals for antipyretics, the specific antipyretics advised for children, and the potential risks associated with combining honey and acetaminophen. Consistent with our findings of limited knowledge regarding dosing intervals for OTC antipyretics, a study conducted from 2012 to 2016, which used an internet panel diary to analyze one-week usage of acetaminophen medications, showed that 85% of participants were aware of the maximum one-time OTC antipyretics dose, while only 47% knew the minimum interval between doses (30). These findings indicate a lack of understanding about the dosing intervals and potential risks associated with antipyretic medications. Therefore, it is imperative to increase education and awareness about the safe and effective use of antipyretic drugs.

This study found that a significant proportion of parents had a positive attitude toward receiving education from physicians about antipyretics, with 61.33% indicating a very positive attitude and 31.19% indicating a positive attitude. Consistent with these findings, a cross-sectional study in Palestine revealed that physician instruction was the most common factor influencing the frequency of medication administration for fever management (31). Furthermore, a recent study demonstrated that providing medication education on dosing safety for liquid acetaminophen and ibuprofen at the time of emergency department discharge significantly improved parents’ knowledge of safe dosing. This led to a 58% increase in correctly identifying a safe dose for their child during the first follow-up call, highlighting the crucial role of education in enhancing knowledge and practice regarding antipyretics (32).

In this study, 49.96% of participants strongly preferred and 28.27% preferred physical cooling methods over antipyretics for fever management. Similarly, an Italian study found that a high percentage of parents (77.8%) and pediatricians (78.5%) used physical techniques such as tepid sponging or cold compresses to alleviate fever (25). However, these physical approaches may not effectively reduce the child’s temperature and can sometimes lead to discomfort (33). In contrast, a study in New Orleans, Louisiana, USA, showed that 94.9% of parents preferred medications, primarily acetaminophen and ibuprofen, to treat fever, with only a few mentioning physical measures (34). The differences in fever management preferences among parents can be attributed to various factors, including cultural practices, access to healthcare and medications, education and awareness, healthcare providers’ recommendations, and personal experiences.

Pearson correlation analysis was used to examine the relationships between knowledge, attitudes, and practices. The results showed that knowledge was positively correlated with practice but negatively correlated with attitude. Attitude was negatively correlated with practice. The negative correlations could have various explanations. As individuals gain more knowledge, they might become more critical or skeptical, leading to a decrease in positive attitudes. Additionally, a higher attitude level might not always translate into better practice (35). Individuals with strong attitudes may face barriers or not feel the need to engage in practice frequently. Similarly, in a study on fever awareness and management practices among parents in urban India, a novel inverse correlation was found between knowledge of available pediatric antipyretics and parental fever management practices (36). This is plausible since parents with limited knowledge are likely to consult doctors for appropriate fever management and follow prescribed drugs and schedules. Further research or context-specific information would be required to better understand the reasons behind these negative correlations.

The results showed that there was a significant increase in knowledge and practice scores related to the use of antipyretics after policy change. This suggests that participants had a better understanding of, and were more likely to apply, proper antipyretic usage after that date. Furthermore, we found that participants responding after January 8th had a higher likelihood of better decision-making. This may be attributed to various factors, such as increased awareness of the appropriate use of antipyretics and improved access to healthcare information. Overall, these findings suggest that the termination of COVID-19 as a Class A infectious disease in China had a positive impact on knowledge, practices, and decision-making related to the use of antipyretics, highlighting the importance of public health interventions in improving healthcare practices during and after pandemics.

This study has several limitations, including a relatively small sample size of 481 valid responses and being conducted in a single city, which may affect the generalizability of the findings. The use of self-reported data could introduce recall bias and social desirability bias. The impact of cultural factors on antipyretic knowledge, attitudes, and practices was not explored. Although the study identified associations between demographic and occupational factors and KAP scores, causation cannot be inferred due to its cross-sectional design. Furthermore, the factors contributing to the negative correlations between knowledge, attitudes, and practices were not investigated. Additional research is required to elucidate the reasons for these negative correlations and to develop effective interventions to enhance antipyretic knowledge, attitudes, and practices.

Overall, fever patients demonstrated moderate levels of knowledge, negative attitudes, proactive precautions for medication administration practices, and moderate decision-making practices. However, following the policy change, a significant improvement was observed in terms of knowledge pertaining to medication administration precautions and decision-making among fever patients. Healthcare providers should develop educational interventions, particularly for suburban residents, and the policy change may influence patients’ knowledge, attitudes, and practices.

## Data availability statement

The original contributions presented in the study are included in the article/supplementary material, further inquiries can be directed to the corresponding author.

## Ethics statement

Therefore been performed in accordance with the ethical standards laid down in the 1964 Declaration of Helsinki (Revised in 2013). The study was approved by the Medical Ethics Committee of the People’s Hospital of Dongxihu District ([2022] No. 40), Wuhan, Hubei, and all participants provided informed consents before completing the questionnaire. All methods were carried out in accordance with relevant guidelines and regulations. The studies were conducted in accordance with the local legislation and institutional requirements. The participants provided their written informed consent to participate in this study.

## Author contributions

YZ: Conceptualization, Formal analysis, Investigation, Methodology, Resources, Writing – original draft, Writing – review & editing. SL: Investigation, Resources, Writing – original draft, Writing – review & editing, Data curation, Supervision, Validation, Visualization. TZ: Validation, Writing – original draft, Writing – review & editing, Conceptualization, Formal analysis, Funding acquisition, Methodology.

## Footnotes

^1^ https://www.wjx.cn
